# Complete Genome Sequence of a Staphylococcus aureus Isolate from a Nasopharyngeal Swab from a Mine Worker in South Africa

**DOI:** 10.1128/MRA.01075-19

**Published:** 2019-12-12

**Authors:** Ashika Singh-Moodley, Mushal Allam, Husna Ismail, Olga Perovic, Wilhelmina Strasheim, Senzo Mtshali, Arshad Ismail

**Affiliations:** a Centre for Healthcare-Associated Infections, Antimicrobial Resistance and Mycoses, National Institute for Communicable Diseases, National Health Laboratory Service, Johannesburg, South Africa; b University of the Witwatersrand, Johannesburg, South Africa; c Sequencing Core Facility, National Institute for Communicable Diseases, National Health Laboratory Service, Johannesburg, South Africa; University of Maryland School of Medicine

## Abstract

Here, we present the complete genome sequence of Staphylococcus aureus NP66, isolated from a South African mine worker.

## ANNOUNCEMENT

Staphylococcus aureus is a Gram-positive, facultative anaerobic bacterium (1) and a major pathogen causing both nosocomial and community-acquired infections. S. aureus has a wide spectrum of clinical manifestations, including wound infections, pneumonia, septicemia, and endocarditis (2, 3). Here, we report the full genome sequence of S. aureus NP66 recovered from a nasopharyngeal swab collected from a male mine worker during an outbreak of Panton-Valentine leukocidin-producing methicillin-susceptible S. aureus infection in a gold mine in South Africa. Ethical clearance was obtained from the University of the Witwatersrand Human Research Committee (protocol number M10464).

The nasopharyngeal swab obtained from the mine worker was cultured as per standard microbiological procedures. For the recovery of bacterial colonies, the swab was subcultured onto 5% horse blood, MacConkey, and/or Chapman’s salt agar plates (Diagnostic Media Products [DMP], National Health Laboratory Service [NHLS], South Africa) and incubated overnight at 37°C. Bacterial colonies were subcultured onto fresh 5% horse blood agar plates (DMP, NHLS) and identified to the species level using matrix-assisted laser desorption ionization–time of flight (MALDI-TOF) mass spectrometry (Bruker Daltonik, Bremen, Germany). Overnight bacterial cultures were started from single colonies and grown at 37°C. The genomic DNA was extracted using the QIAamp DNA minikit (Qiagen, Germany). The PacBio library was produced using a SMRTbell template prep kit 1.0, and a single SMRTbell 10-kb library was prepared according to the protocol “Preparing SMRTbell Libraries Using PacBio Barcoded Adapters for Multiplex SMRT Sequencing” (Pacific Biosciences, Menlo Park, CA, USA). The genome was sequenced using a single-molecule real-time (SMRT) cell with the PacBio Sequel sequencing platform, following the manufacturer’s specifications, with on-plate concentration of 4 pM using Sequel chemistry V2 and a 10-hour movie time. Sequencing yielded 765,268,787 bases from 316,295 reads, with an average length of 2,419 bp. The quality of the reads was determined and analysis of the data was performed using PacBio SMRT link version 5.1. The generated subreads were assembled using Hierarchical Genome Assembly Process version 4 (HGAP4) followed by circularization using Berokka (https://github.com/tseemann/berokka) and annotation using the NCBI Prokaryotic Genome Annotation Pipeline (PGAP) (4). The Comprehensive Antibiotic Resistance Database (CARD) (5), PlasmidFinder (6), the Virulence Factor Database (VFDB) (7), and *spa*Typer version 1 (8) were used to predict the resistance genes, plasmids, virulence genes, and *spa* type, respectively. Multilocus sequence typing (MLST) was determined using mlst (https://github.com/tseemann/mlst). Default settings were used in all software unless otherwise noted.

A total of 295,201 filtered subreads were generated after sequencing (average subread length, 2,400 bp; subread *N*_50_, 2,898 bp; 249-fold coverage). The reads were assembled, and we found that the complete genome sequence of S. aureus NP66 consists of 2,752,396 bp with a G+C content of 32.9%, including 2,703 protein-coding genes, 78 pseudogenes, and 82 RNA genes (19 rRNAs, 59 tRNAs, and 4 noncoding RNAs). Using the CARD database (5), we found 11 antibiotic resistance genes in this genome, including the beta-lactamase resistance gene (*blaZ*, 94.66% identity), the fosfomycin resistance gene (*fosB3*, 99.28% identity), and others, such as genes encoding the two-component system ArlS-ArlR, which plays a role in capsule production (*arlR*, 100% identity; *arlS*, 100% identity), and genes encoding efflux pumps (*LmrS*, 99.38% identity; *mepR*, 100% identity; *mgrA*, 100% identity; *norA*, 100% identity). The isolate was negative for the Panton-Valentine leukocidin exotoxin but did harbor 75 potential genes associated with virulence factors. Furthermore, no plasmids were present. The *spa* type was t909, and MLST analysis revealed that the isolate belonged to sequence type 12 (ST12).

### Data availability.

The complete genome sequence of NP66 has been deposited in DDBJ/ENA/GenBank under the accession number CP041037. The raw reads have been submitted to the SRA under BioProject accession number PRJNA548666 and SRA accession number SRR9879543.
